# Annual acknowledgement of manuscript reviewers

**DOI:** 10.1186/2045-4015-3-4

**Published:** 2014-01-23

**Authors:** Bruce Rosen, Avi Israeli

**Affiliations:** 1 Israel Journal of Health Policy Research (IJHPR), London, UK

## Abstract

**Contributing reviewers:**

The Editors of *Israel Journal of Health Policy Research* would like to thank all reviewers, both external and Editorial Board Members, who have contributed to the journal in 2013, and whose valuable support continues to be essential to the success of the journal.

## 

Linda Aiken

United States of America

Eric Amster

Israel

Shai Ashkenazi

Israel

Toni Ashton

New Zealand

Yaacov Baruch

Israel

Netta Bentur

Israel

Yitshal Berner

Israel

Nina Bickell

United States of America

Jeffrey Borkan

United States of America

Ivy Bourgeault

Canada

Barbara Braun

United States of America

Mayer Brezis

Israel

Jenny Brodsky

Israel

Susan Busch

United States of America

Yeezkel Caine

Israel

David Chinitz

Israel

Paul Cleary

United States of America

Robert Cohen

Jordan

Antonia Cronin

United Kingdom

Gordon DeFriese

United States of America

Milka Donchin

Israel

Amy Eisenstein

United States of America

Aharon Finestone

Israel

Gary Freed

United States of America

Nurit Friedman

Israel

Paul Froom

Israel

Stanton Glantz

United States of America

Shimon Glick

Israel

Rosa Gofin

United States of America

Itamar Grotto

Israel

Pinchas Halpern

Israel

Paul Hansen

New Zealand

Naomi Hausman

Israel

Donna Havens

United States of America

Anthony Heymann

Israel

Dominic Hodgkin

United States of America

Giora Kaplan

Israel

Arthur Kellermann

United States of America

Alex Kemper

United States of America

Sun-Young Kim

United States of America

Richard Kravitz

United States of America

Fleur-Ange Lefebvre

Canada

Moshe Leshno

Israel

Robert Like

United States of America

Michelle Macy

United States of America

Orly Manor

Israel

Gregory Marchildon

Canada

Rob McNeill

New Zealand

David Mirvis

United States of America

Shlomo Mor-Yosef

Israel

Susan Murray

Canada

Yehuda Neumark

Israel

Nurit Nirel

Israel

Ora Paltiel

Israel

Kobi Peleg

Israel

Joseph Pliskin

Israel

Avi Porath

Israel

Basil Porter

Israel

Shmuel Reis

Israel

Linda Resnik

United States of America

Shmuel Rishpon

Israel

Laura Rosen

Israel

Alan Rosenstein

United States of America

Brendan Saloner

United States of America

Richard Saltman

United States of America

Jörg Schaaber

Germany

Gillian Schiller

United Kingdom

Harald Schmidt

United States of America

Michael Schoenbaum

United States of America

Stephen Schoenbaum

United States of America

Chen Shapira

Israel

Amir Shmueli

Israel

Pierre Singer

Israel

Daniel Sperling

Israel

Katherine Swartz

United States of America

Orna Tal

Israel

Shlomo Vinker

Israel

Michael Weingarten

Israel

Rachel Wilf Miron

Israel

Jenny Wright

United Kingdom

Yael Yishai

Israel

Eyal Zimlichman

Israel

Amitai Ziv

Israel

Jack Zwanziger

United States of America

